# Scale development and an educational program to reduce the stigma of schizophrenia among community pharmacists: a randomized controlled trial

**DOI:** 10.1186/s12888-021-03208-z

**Published:** 2021-04-26

**Authors:** Tomoo Fujii, Manako Hanya, Kenta Murotani, Hiroyuki Kamei

**Affiliations:** 1 Office of Clinical Pharmacy Practice and Health Care Management, Faculty of Pharmacy, Meijo University, Nagoya, Aichi Japan; 2 Biostatistics Center, Graduate School of Medicine, Kurume University, Kurume, Fukuoka Japan

**Keywords:** Pharmacist, Attitude, Contact-based educational programs, Stigma scale, Schizophrenia

## Abstract

**Background:**

Stigma associated with mental disorders is rooted among many pharmacists, and represents a major barrier to patient support in community-based psychiatry. We developed an assessment scale that is specifically designed to assess the level of stigma that pharmacists may have toward schizophrenia, and then examined the effects of reducing stigma with an educational program that focuses on communication with patients diagnosed with schizophrenia (PDS) using the newly developed Stigma Scale towards Schizophrenia for Community Pharmacists (SSCP).

**Methods:**

SSCP was developed by exploratory factor analysis with promax rotation based on responses from 822 randomly selected community pharmacists. Furthermore, a randomized controlled trial was conducted for 115 community pharmacists to clarify the effects of reducing the stigma of schizophrenia using an educational program for them with a focus on communication with PDS. Participants were individually allocated to two groups: educational lecture group (56; only attending a lecture on schizophrenia) or contact-based intervention group (59; communicating with PDS and attending the lecture). The stigma assessment using SSCP was conducted immediately before and after the educational intervention.

**Results:**

A total of 4 factors and 27 items were extracted from the exploratory factor analysis to comprise the SSCP. Cronbach’s α of SSCP, social distance at professional pharmacy service (factor I), attitudes towards PDS (factor II), self-disclosure (factor III), and social distance in personal (factor IV) were 0.89, 0.88, 0.76, 0.62, and 0.62, respectively. Educational program-related changes of the median (interquartile range) total SSCP score from baseline were − 9.0 (− 16.0 – − 5.0) in the contact-based intervention group and − 3.0 (− 7.0–1.0) in the educational lecture group, reflecting a significant reduction of stigma levels in the contact-based intervention group. On examining the SSCP subscales, scores for factor I and factor II significantly improved. The educational program was more effective for pharmacists aged 20–39 years or with negligible experience of communicating with PDS at work and/or in private life.

**Conclusions:**

SSCP and the educational program for community pharmacists that focuses on communication with PDS were useful for assessing and reducing, respectively, the stigma attached by these pharmacists to schizophrenia.

**Trial registration:**

UMIN Clinical Trials Registry (UMIN000043189, registered on January 30, 2021), Retrospectively registered.

## Background

The number of people with mental disorders in Japan exceeded 3.9 million in 2014 and is steadily increasing [1]. Patients diagnosed with schizophrenia account for nearly 50% of all long-term hospitalized patients (≥1 year), and concerns have been expressed over their prolonged hospitalization [1]. The Ministry of Health, Labor and Welfare clarified its vision to promote a shift in mental healthcare from inpatient treatment to community life support in 2004 [2]. Therefore, pharmacists are increasingly expected to provide appropriate medical information and increase the quality of psychiatric services for mentally ill patients. However, a negative stereotype or stigma of mental disorders is rooted among many pharmacists [3, 4] and other healthcare professionals [5, 6] and represents a major barrier to patient support in community-based psychiatry. The stigma attached to mental disorders, specifically schizophrenia, is stronger in Japan than in other countries [7]. Until 2002, the Japanese term for schizophrenia was “seishin bunretsu byo”, which means “split-mind illness”. The negative connotations associated with this name had made clinicians largely reluctant to use the term when communicating with patients and their families [8]. In 2002, as a way to reduce the stigma caused by the term “seishin bunretsu byo”, a change in naming was decided. The new term for schizophrenia became “togo shitcho sho”, meaning an “illness of integration loss”. While a renaming of the condition may prevent certain negative connotations triggered by the old name, a linguistic change alone without a new conception of the condition itself is probably inadequate to decrease stigma [9, 10]. Patients should be diagnosed based on their individual recovery processes, as well as symptoms, after being completely overwhelmed by illnesses to lead a meaningful life despite ongoing symptoms and mental challenges.

Community pharmacists play an important role in the care of patients diagnosed with schizophrenia who require pharmacological treatments because they provide guidance on the effects and side effects of medication as well as support for continued adherence to medication [11]. However, stigma among pharmacists towards patients diagnosed with schizophrenia may interfere with adherence and worsen symptoms if patients discontinue their medication [3, 12]. In our previous internet survey on stigmatic attitudes towards the mentally ill, involving 870 physicians, nurses, pharmacists, and general citizens in Japan, the stigma-related scores of pharmacists were similar to those of physicians and nurses, and they had relatively positive attitudes towards the mentally ill [13]. On the other hand, in comparisons of pharmacists and general citizens, the recognition of mental illness was more positive in the former, but the social distance from these patients was similar between these groups, revealing the need to reduce this distance and decrease stigma among pharmacists [13]. Social distance refers to attitudes that prevent social participation by people with mental disorders, namely, psychological distance from the patients, and is a concept considered to be one of the important components of mental health-related stigma [14]. This survey also suggested the effectiveness of appropriate communication with patients through working/training in psychiatric facilities to reduce social distance from people with mental disorders [5, 13]. In order to decrease the stigma of schizophrenia, social distance needs to be reduced and more positive attitudes have to be promoted towards these patients. Therefore, combining educational interventions based on knowledge of schizophrenia and appropriate communication with patients, rather than awareness-enhancing approaches, such as educational lectures alone, is considered to be more effective [15, 16].

Educational programs for pharmacists, with a focus on communicating with patients, may be important for reducing stigma. However, the effectiveness of contact-based educational programs for pharmacists to reduce the stigma of schizophrenia has not yet been demonstrated.

We previously employed stigma scales to measure negative attitudes towards mental disorders and social distance manifested as avoidance responses to the mentally ill in society [17, 18]. To confirm the effectiveness of educational programs for pharmacists, stigma scales that accurately simulate actual settings, in which pharmacists manage patients diagnosed with schizophrenia, are essential.

Therefore, a scale to assess the stigma of schizophrenia among pharmacists was developed in the present study and its reliability was verified. A randomized controlled trial was then conducted to confirm the effectiveness of the pharmacist educational program with a focus on communication with patients to reduce the stigma of schizophrenia.

## Methods

The present study was approved by the Meijo University Research Ethics Board to ensure privacy, confidentiality, and anonymity (H30–3). It was conducted according to the principles expressed in the Declaration of Helsinki.

### Development of a scale to assess the stigma of schizophrenia among community pharmacists

To develop a scale to assess the stigma of schizophrenia among community pharmacists, items related to the constructs of stigma as a barrier for pharmacists to provide professional support for patients diagnosed with schizophrenia were extracted from existing scales [5, 14, 19–21]. A pool of items was created with these items and those newly developed upon deliberations among 3 pharmacists (1 university professor conducting research on psychiatry, 1 communication specialist, and 1 researcher). There were 67 items, representing 3 constructs; 1) recognition of patients diagnosed with schizophrenia, 2) social distance, and 3) self-disclosure/help-seeking behavior. The appropriateness of the 67 items in terms of content and expression was then examined with 12 pharmacists, including 2 of the above-mentioned pharmacists and 3 pharmacists specializing in psychiatry. The 12 pharmacists were recruited using a convenience sampling method. They narrowed down 67 items and a 33-item scale was created. The scale was created by eliminating items that did not meet the following criteria: 1) the item represents a stigma that prevents pharmacists from providing service to their patients in an effective manner, 2) the item is suitable as an evaluation criteria for the educational program, and 3) the number of questions is appropriate not to be a burden to subjects.

The assessment tool was distributed with a survey on personal background information to 1500 pharmacies by mail in Aichi Prefecture between December 1, 2018, and January 31, 2019. These pharmacies were randomly selected from those belonging to the Aichi Pharmaceutical Association. One questionnaire was mailed to each of the above 1500 pharmacies and one representative from each pharmacy responded. All 1500 pharmacies received the questionnaire.

A 5-point scale was used in the assessment tool as follows: 1: strongly disagree, 2: disagree, 3: neither agree nor disagree, 4: agree, 5: strongly agree. Negative responses were given 5 points, and positive responses 1 point. Correlations between each item and the total score (item total correlation) were confirmed from the scale proposals collected. Based on the answers provided, an exploratory factor analysis was performed using the principal axis factoring method with a promax rotation to further examine the factors constituting the assessment tool. The 33-item scale created by 12 pharmacists described above was extracted into a 27-item scale by the factor analysis.

Cronbach’s *α* confidence coefficient was calculated for each item and factor of the assessment tool to assess the level of internal consistency. The correlation between the total score and social desirability scale was confirmed from the 27-item scale. The social desirability bias can be defined as the tendency of individuals to report favorable impressions of themselves on measures that ascertain sociological and psychological variables of interest such as attitudes to mental illness. The 27-item scale that we developed may not fully represent the degree of stigma if there was a social desirability bias. In this study, we measured the social desirability bias by the method used by Makita [22, 23]. Specifically, we determined that there was a selection bias if our scale was highly correlated with the total scores of the following 3 items: 1) I do not necessarily like everyone that I know, 2) when I eat at home, I do not behave as well as I do when I am out with others to eat, and 3) I would not feel discontent if I develop schizophrenia. These items require participants to respond to statements using a 5-point Likert scale from “strongly disagree” to “strongly agree”. Each item is scored 1–5, such that scores range from 3 to 15. The test-retest reliability of the 27-item scale was verified by conducting a retest for those who consented among the sub-sample of community pharmacists 8 to 12 weeks after the completion of the survey, and the intra-class correlation comparing the total scores at the 2 points they completed was calculated.

Furthermore, to confirm criterion-related validity, the correlation between the total 27-item scale score and total score from the following 2 conventional scales was used to assess the stigma of mental disorders: the Whatley Social Distance Scale (WSDS) [14] and Index of Attitudes toward the Mentally Ill (IATM) [19]. WSDS examines attitudes that prevent social participation by people with mental disorders (social distance), and IATM measures the levels of negative recognition of these disorders. We used the 2 scales in our previous studies, involving pharmacy students [17], and an internet survey [13], conducted by physicians, nurses, pharmacists, and general citizens. The scale developed through these processes was named the Stigma Scale towards Schizophrenia for Community Pharmacists (SSCP), and was used in subsequent studies.

### Randomized controlled trial

A randomized controlled trial was conducted to clarify the effects of reducing the stigma of schizophrenia using an educational program for community pharmacists with a focus on communication with patients diagnosed with schizophrenia.

### Participants and study design

**Fig. 1:**
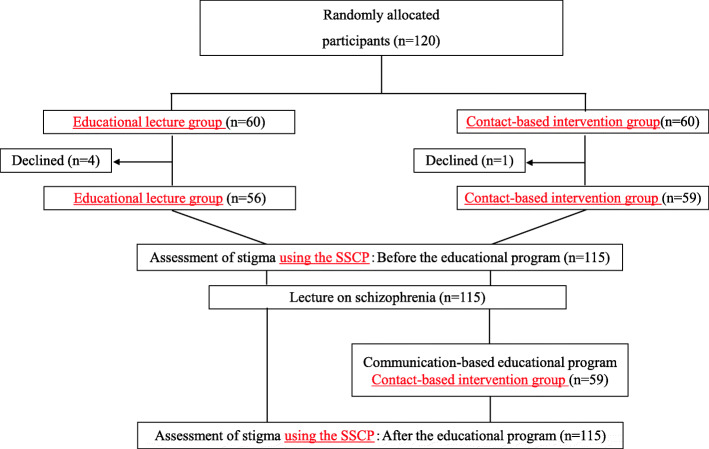
Study design

An outline of the study design is shown in Fig. 1. Community pharmacies in Aichi Prefecture in Japan were randomly selected to receive a document to recruit participants by e-mail or post, and consent was obtained from 120 pharmacists employed by these pharmacies. The 120 pharmacists did not include the 1500 pharmacists recruited for the development of the scale. They participated in the educational program twice, on July 28 and November 10, 2019, and were divided into 2 groups of 60, adopting the stratified block randomization method using a computer-generated randomization list with a block size of four: educational lecture group (only attending a lecture on schizophrenia) and contact-based intervention group (communicating with patients diagnosed with schizophrenia and attending the lecture). Randomization was stratified by (1) sex (female versus male), (2) age (< 30 years versus > 30 years), (3) experience of communicating with people with mental disorders (whether participants have or do not have this experience). Four participants in the educational lecture group withdrew and 1 participant in the contact-based intervention group withdrew, leaving the final number of members in each group to be 56 and 59, respectively (a total of 115 participants). Approximately 50% of each group participated in each session. After the lecture, the group to which each participant belonged was disclosed.

### Outline of the educational program

All 115 participants attended a 60-min lecture on schizophrenia given by a psychiatrist. The contents of the lecture were the disease concept of schizophrenia and the current position of schizophrenia in overall psychiatric disorders, the epidemiology, various symptoms of each patient, effects of the disease on social activities, diagnostic criteria, treatment methods based on the latest evidence, treatment effects, main side effects, and prognosis of schizophrenia. In addition, the lecture highlighted the importance of addressing gaps in treatments, as early diagnosis and intervention have a significant impact on the recovery and prognosis of patients diagnosed with schizophrenia. The lecture also included an emphasis on medication adherence to prevent recurrence and recent strategies to promote adherence such as the use of long-acting injections and options for individuals to select a dosage form that fit their condition and lifestyle.

After the lecture, 59 contact-based intervention members formed groups of 4 or 5 to perform the following activities in a single room:

1. A lecture staff introduction and ice-breaking (self-introduction in each group)
2. A lecture on mental disorders and the associated stigma (prejudice and discrimination)
3. Group work 1: holding a group discussion on the management of patients diagnosed with schizophrenia, and making a presentation with 1 member of each group (a total of 5 groups) as the presenter
4. Group work 2: holding a group discussion and offering opinions on the points of an interview with patients diagnosed with schizophrenia to clarify their experience
5. An interview with patients diagnosed with schizophrenia, who was allocated to the table of each group (a total of 6 people) and introduced him/herself for 20 min using a self-introduction sheet previously filled out
6. Interviews with 2 other people (a total of 3 rotations)
7. Group work 3: holding a group discussion on points of learning by pharmacists from patient experiences, making a presentation with 1 member of each group (a total of 5 groups) as the presenter, followed by a lecture to summarize the opinions offered at the group presentations.

The 6 (4 males and 2 females) patients diagnosed with schizophrenia who shared their experiences belonged to a patient group in Nagoya city. They had taken antipsychotics for 5 years or longer and were visiting psychiatric hospitals as outpatients. Their signed consent was previously obtained using a written document specifying the study objective.

After the consent process, they entered: 1) medical history, 2) difficulties associated with pharmacotherapy, 3) issues they may or may not consult about with community pharmacists, 4) cases in which they had perceived stigmatizing behaviors/attitudes (prejudice and discrimination), and 5) demands to be fulfilled by community pharmacists, in a self-introduction sheet, and rehearsed self-introductions using this sheet.

### Stigma assessment

The stigma of schizophrenia among community pharmacists was assessed using SSCP at 3 points: before the lecture (both groups: T1), immediately after the lecture (educational lecture group: T2), and immediately after communicating with patients diagnosed with schizophrenia (contact-based intervention group: T3). SSCP consists of 27 statements to be evaluated on a 5-point scale: <Strongly disagree>, <Disagree>, < neither agree nor disagree >, <Agree>, and, <Strongly agree> which were scored as 1–5, respectively. Five items among 27 statements were reverse scored (i.e., #11, 20, 24, 25, and 26). The total score ranged between 27 and 135. Scores that were lower than or equal to a median of 81 represented more favorable attitudes.

### Statistical analysis

Statistical analyses were performed using IBM SPSS statistics ver. 22. To identify fundamental factors from the 33-item scale, exploratory factor analysis was performed using the principal axis factoring method with a promax rotation. The choice of the number of factors was based on the scree plot. Items with factor loadings lower than 0.4 were deemed meaningful and assigned to the given factor, with only the highest factor loading for each item being considered. Even if an item had a factor load of lower than 0.4, the item was adopted if researchers found it necessary to explain the construct to which the item belongs. We labeled each factor based on what best characterized the group of items that loaded on a particular factor. The internal consistency of the SSCP and subscales was evaluated using Cronbach’s *α* coefficient and criterion-related validity for SSCP was examined using Spearman’s correlation (*rs*). In the randomized controlled trial section, the attributes of the 2 groups were compared using the Mann-Whitney U test and chi-squared test. The Wilcoxon signed-rank test was used to compare after the educational program. Differences in the effectiveness of the educational program in the 2 groups were compared using the Mann-Whitney U test. The effect size *r* was calculated using the standardized test statistic (*Z*) and sample size (*N*) (*r = Z/√N*). An effect size of 0.1 was considered to be small, 0.3 medium, and 0.5 or greater a large effect [24]. The significance of differences was set at two-tailed *p* < 0.05 unless otherwise specified.

## Results

### Testing of the 33-item scale to assess reducing stigma

#### Response rate and respondent characteristics

There were 822 responses (response rate: 54.8%) and 806 were valid for analysis (valid response rate: 53.7%). The mean age of respondents was 42.3 ± 12.1 years. There were 490 (60.8%) males and 316 (39.2%) females. Their mean length of pharmacy experience was 15.6 ± 10.4 years.

#### Item-total correlations

An item-total correlation of 0.2 to 0.5 is considered to be the most appropriate [20]. None of the 33 statements showed an item-total correlation of lower than 0.2.

#### Exploratory factor analysis

Table 1 shows factorial patterns after the promax rotation, inter-factor correlations, Cronbach’s *α* coefficient, and the median (inter-quartile range) for each item. Prior to the factor analysis, the mean score and standard deviation for each item were calculated to confirm the absence of a ceiling or floor effect. In the Kaiser-Meyer-Olkin measure of sampling adequacy, it is recommended that a range of 0–1 be allowed and values need to be higher than 0.6. The factor model showed a coefficient of 0.91, confirming its sufficient sampling adequacy.

**Table 1:** Factorial patterns after the promax rotation for community pharmacists

27 items, ***α*** = 0.89	Extraction factor					
	I	II	III	IV	Median (inter-quartile range)	
**Factor I: Social distance at professional pharmacy service,** ***α*** **= 0.88**						
1. If possible, I would rather avoid administering and advising about medication for patients with schizophrenia.	**0.89**	−0.11	− 0.03	0.03	2.0 (2.0–3.0)	
2. If possible, I would rather avoid consultations with patients with schizophrenia as much as possible.	**0.78**	−0.08	0.04	−0.03	2.0 (2.0–3.0)	
3. If possible, I would rather avoid home visits for patients with schizophrenia.	**0.73**	−0.10	0.04	0.04	3.0 (2.0–4.0)	
4. I feel that it is too much work to deal with patients with schizophrenia.	**0.72**	0.09	−0.11	−0.02	3.0 (2.0–3.0)	
5. I would rather be involved in care for patients with physical illnesses than those with schizophrenia.	**0.63**	0.08	−0.11	− 0.01	3.0 (2.0–3.0)	
6. I am afraid of administering and advising about medication for patients with schizophrenia.	**0.58**	0.01	0.02	0.05	2.0 (2.0–3.0)	
7. If a patient hands me a prescription that includes medications for schizophrenia, I would try to avoid discussing his/her illness as much as possible.	**0.55**	0.03	0.18	−0.17	3.0 (2.0–4.0)	
8. I find it difficult to deal with patients with schizophrenia, particularly during busy hours.	**0.55**	0.18	0.03	−0.18	3.0 (2.0–4.0)	
9. Despite my principles as a health care provider, I react negatively to patients with schizophrenia.	**0.53**	0.19	−0.03	0.04	2.0 (2.0–3.0)	
10. I find it difficult to communicate with patients with schizophrenia.	**0.52**	0.08	0.07	−0.06	3.0 (2.0–4.0)	
11. I am not worried about dealing with situations in which I receive prescriptions or medication records for schizophrenia medications from patients. (R)	**0.47**	− 0.17	−0.02	0.18	2.0 (2.0–3.0)	
**Factor II: Attitudes towards patients diagnosed with schizophrenia,** ***α*** **= 0.76**						
12. I think that schizophrenia affects the daily lives of patients.	−0.05	**0.64**	0.02	−0.00	2.0 (2.0–3.0)	
13. I do not think that patients can recover from schizophrenia.	−0.01	**0.58**	−0.01	0.08	2.0 (2.0–3.0)	
14. I think that patients with schizophrenia are not capable of understanding their own illness.	0.02	**0.55**	0.04	−0.07	2.0 (2.0–3.0)	
15. I think that patients with schizophrenia are always suffering from symptoms that include hallucination and delusion.	−0.08	**0.54**	−0.03	−0.00	2.0 (2.0–3.0)	
16. I think that patients with schizophrenia are not capable of understanding and adhering to the suggested treatment regimen.	0.00	**0.53**	0.04	0.05	2.0 (2.0–3.0)	
17. I think that more than 50% of patients with schizophrenia are not working hard enough to improve their own conditions.	0.09	**0.44**	−0.08	−0.05	2.0 (2.0–3.0)	
18. I think that patients with schizophrenia have difficulties reintegrating into society.	0.04	**0.43**	0.04	0.11	3.0 (2.0–3.0)	
19. I think that patients with schizophrenia are dangerous.	0.19	**0.35**	−0.01	0.11	3.0 (2.0–3.0)	
**Factor III: Self-disclosure,** ***α*** **= 0.62**						
20. Unlike other diseases, if I had schizophrenia, I would be able to tell my friends about it. (R)	0.06	−0.14	**0.58**	0.11	3.0 (2.0–4.0)	
21. Unlike other diseases, if I had schizophrenia, I would not be easily able to tell my family about it.	0.08	0.03	**0.57**	0.05	2.0 (2.0–3.0)	
22. Unlike other diseases, I would not be able to tell my colleagues that I was being treated for schizophrenia.	0.07	−0.02	**0.57**	−0.03	4.0 (3.0–4.0)	
23. Unlike other diseases, if I had schizophrenia, I would hesitate to seek help from health professionals.	−0.03	0.17	**0.42**	−0.11	2.0 (2.0–3.0)	
**Factor IV: Social distance in personal,** ***α*** **= 0.62**						
24. If a colleague of mine told me that he/she has schizophrenia that has been well-managed by medications, I would still be able to work with him/her without any issues. (R)	−0.04	− 0.00	− 0.00	**0.66**	2.0 (2.0–3.0)	
25. If a candidate has the most appropriate skills for the job, employers should hire a patient whose symptoms of schizophrenia are well-managed by medications. (R)	−0.14	0.06	0.03	**0.55**	2.0 (2.0–3.0)	
26. I would not mind if a patient with schizophrenia lived next door. (R)	0.22	0.01	−0.06	**0.40**	3.0 (2.0–4.0)	
27. I would not want my children to work with a patient with schizophrenia even if his/her symptoms are well-managed by medications.	0.19	0.13	0.10	**0.36**	2.0 (2.0–3.0)	
Factor correlation	1.00	.59	.42	.38		
	.59	1.00	.37	.42		
	.42	.37	1.00	.29		
	.38	.42	.29	1.00		

*n* = 806. (R): reverse scored. The values for Factor I to IV represent factor loading

Significant results (*χ2* = 6578.2, *df* = 351, *p* < 0.001) confirming the appropriateness of this model were also obtained from Bartlett’s test for sphericity. Following the examination of all 33 items through an exploratory factor analysis, 4 factors and 27 items were selected. The cumulative proportion of variance explained was 45.8%. Six items with a factor loading lower than 0.4 were removed. The 6 items removed were as follows: “When patients with schizophrenia present with physical symptoms (e.g. nausea, back pain, headache), I might think that they are manifested because of mental issues”, “I feel that there is nothing I can do for patients with schizophrenia in terms of their recovery”, “I cannot understand the behavior of patients with schizophrenia that is caused by hallucinations and delusions”, “I think I can actively identify specific problems that patients with schizophrenia may have”, “I cannot be friends with a patient with schizophrenia”, and “If I had schizophrenia and was not able to control the symptoms myself, I would consider myself a weak person”.

However, items judged to be necessary for explaining the components were not excluded, even if factor loading was lower than 0.4. The 4 factors were named as follows: factor I; social distance at professional pharmacy service, factor II; attitudes towards patients diagnosed with schizophrenia, factor III; self-disclosure, and factor IV; social distance in personal. The median (inter-quartile range) of the total score was 70.0 (63.0–78.0) for the entire scale, 29.0 (25.0–34.0) for factor I, 19.0 (17.0–22.0) for factor II, 11.0 (10.0–12.0) for factor III, and 10.0 (9.0–12.0) for factor IV. These scores indicated a relatively positive response because they were lower than 50% of the total score.

### Testing of the 27-item scale to assess reducing stigma

#### Internal consistency

Cronbach’s *α* was 0.89 for the entire scale, 0.88 for <factor I: social distance at professional pharmacy service >, 0.76 for <factor II: attitudes towards patients diagnosed with schizophrenia >, 0.62 for both <factor III: self-disclosure> and < factor IV: social distance in personal >.

#### Test-retest reliability

Among the initial samples, a subset of 81 consented to the retest. The intra-class correlation between the total scores at the 2 points was 0.90 (95% CI 0.84–0.93, *p* < 0.001), exceeding 0.7, and, thus, the value confirmed sufficient reliability.

#### Social desirability bias

No correlation was observed between the total SSCP score of 806 pharmacists and the total social desirability scale (*rs* = − 0.117, *p* = 0.213).

#### Criterion-related validity

The correlation between the total SSCP score and total WSDS/IATM scores was calculated. Total score correlations between SSCP and WSDS or IATM were 0.58 (*p* < 0.001) and − 0.62 (*p* < 0.001), respectively.

### Randomized controlled trial to assess effects of contact-based educational programs on educational lectures alone

No significant differences were observed in participant backgrounds between the 2 groups (Table 2).

**Table 2:** Baseline characteristics of participants in a randomized controlled study

Characteristics	Educational lecture group	Contact-based intervention group	
	*n* = 56	*n* = 59	*p*
Gender, %female	41.1	42.4	0.89
Age, years: mean ± S.D.	36.8 ± 8.9	37.7 ± 10.0	0.82
Age group: %			
20–29	26.8	28.8	0.93
30–39	37.5	32.2	
40–49	23.2	23.7	
50<	12.5	15.2	
Pharmacist, years: mean ± S.D.	10.3 ± 6.9	11.9 ± 9.3	0.70
Psychiatric work experience, %positive	30.4	25.4	0.56
Experience of mental illness via a family member or close friend, %positive	32.1	32.2	0.99
Experience of schizophrenia via a family member or close friend, %positive	12.5	10.2	0.69
Frequency of medication counseling for schizophrenia patients, %positive			
Almost every day	0	1.7	0.37
A few patients a week	12.5	11.9	
A few patients a month	42.9	39.0	
A few patients a year	21.4	32.2	
Nothing	23.2	15.3	
SSCP, median (inter-quartile range)	74.0 (67.0–82.0)	71.0 (66.0–80.0)	0.276
Factor I, median (inter-quartile range)	32.0 (29.0–37.0)	31.0 (26.0–36.0)	0.242
Factor II, median (inter-quartile range)	20.0 (18.0–22.0)	20.0 (18.0–22.0)	0.840
Factor III, median (inter-quartile range)	12.0 (9.0–14.0)	11.0 (9.0–13.0)	0.336
Factor IV, median (inter-quartile range)	10.0 (9.0–11.8)	10.0 (8.0–12.0)	0.292

Statistical analysis was performed using the chi-squared test or Mann-Whitney U test

### Effects of the contact-based educational program on reducing stigma

In the contact-based intervention and educational lecture groups, the total SSCP score and factor I, II, and III scores after the educational program were significantly better than the baseline score (Table 3). Educational program-related changes in the median (inter-quartile range) total SSCP score were − 9.0 (− 16.0 – − 5.0) in the contact-based intervention group and − 3.0 (− 7.0–1.0) in the educational lecture group (improvement rate, 15.5 and 5.2%, respectively; *p* < 0.001) (Table 3).

**Table 3:** SSCP score before and after the intervention

	Educational lecture group				Contact-based intervention group					
	*n* = 56				*n* = 59					
	Baseline	Post-test	*p*^a^	Difference	Baseline	Post-test	*p*^a^	Difference	*p*^b^	Effect size
SSCP, median (inter-quartile range)	74.0 (67.0–82.0)	69.0 (64.3–77.8)	< 0.001	−3.0 (−7.0–1.0)	71.0 (66.0–80.0)	62.0 (54.0–67.0)	< 0.001	−9.0 (−16.0−−5.0)	< 0.001	0.41
Factor I, median (inter-quartile range)	32.0 (29.0–37.0)	29.5 (26.0–35.0)	< 0.001	−1.0 (− 5.0–0)	31.0 (26.0–36.0)	25.0 (22.0–30.0)	< 0.001	−4.0 (−9.0−−2.0)	0.001	0.32
Factor II, median (inter-quartile range)	20.0 (18.0–22.0)	18.0 (17.0–21.8)	0.02	−1.0 (− 2.8–1.0)	20.0 (18.0–22.0)	16.0 (12.0–18.0)	< 0.001	−5.0 (−7.0−−2.0)	< 0.001	0.49
Factor III, median (inter-quartile range)	12.0 (9.0–14.0)	11.0 (9.0–13.0)	0.01	−1.0 (−2.0–0)	11.0 (9.0–13.0)	10.0 (8.0–12.0)	0.04	−1.0 (−2.0–1.0)	0.94	0.01
Factor IV, median (inter-quartile range)	10.0 (9.0–11.8)	10.0 (9.0–11.0)	0.52	0.0 (−1.0–1.0)	10.0 (8.0–12.0)	9.0 (8.0–10.0)	0.35	0.0 (−1.0–1.0)	0.18	0.13

^a^: vs. each baseline

^b^: vs. Lecture group

Factor I: social distance at professional pharmacy service

Factor II: attitudes towards patients diagnosed with schizophrenia

Factor III: self-disclosure

Factor IV: social distance in personal

Statistical analysis was performed using the Wilcoxon signed-rank test or Mann-Whitney U test

The improvement rates for each factor in the contact-based intervention and educational lecture groups were as follows: factors I: 18.3 and 7.1%, II: 23.0 and 4.5%, III: 5.0 and 6.7%, and IV: 1.0 and 0%, respectively, revealing marked improvements in scores for factors I and II (*p* = 0.001 and *p* < 0.001, respectively). The effect sizes associated with the contact-based educational session were as follows: entire SSCP: 0.41, factors I: 0.32 and II: 0.49, respectively, revealing a moderate difference among both groups.

### Effects of demographic characteristics on differences in stigma between 2 groups

Table 4 shows the effects of demographic characteristics on differences in stigma between the 2 groups. Among participants in their 40s and older or who had experience of schizophrenia via family members or close friends or provide medication counseling for a few patients diagnosed with schizophrenia each week, no additional effect of the contact-based educational intervention on the educational lecture was observed. However, in all subgroups other than that described above, participants in the contact-based intervention group showed a significantly greater change than those in the educational lecture group.

**Table 4:** Effects of demographic characteristics on differences in the stigma for changes in SSCP scores

Characteristics	Educational lecture group	Contact-based intervention group	
	*n* = 56	*n* = 59	*p*
Gender			
Female	−2.0 (− 7.0–1.0)	−10.0 (− 21.0−− 7.0)	< 0.001
Male	− 3.0 (−8.0–1.0)	− 8.0 (− 14.5−− 4.0)	0.008
Age			
20–29	−3.0 (− 7.0–0.0)	− 12.0 (− 21.0−− 7.0)	0.006
30–39	− 2.0 (− 6.5–2.0)	− 9.0 (− 21.0−− 3.0)	0.007
40–49	−6.0 (− 12.0–1.5)	−7.5 (− 9.3−− 4.0)	0.616
50<	−3.0 (−7.0−− 2.0)	−13.0 (− 16.0−− 7.5)	0.071
Has visited a mental hospital			
positive	− 3.0 (− 4.5–2.5)	−8.0 (−15.5−− 4.5)	0.007
negative	−3.0 (−8.0–1.0)	−9.0 (− 17.0−− 5.0)	< 0.001
Experience of mental illness via a family member or close friend			
positive	−1.5 (−6.5–2.3)	−9.0 (−17.0−−8.0)	0.001
negative	−3.0 (−7.0–0.3)	−8.5 (−16.0−−4.0)	0.003
Experience of schizophrenia via a family member or close friend			
positive	1.0 (−7.0–3.0)	−8.0 (−13.0−−3.8)	0.10
negative	−3.0 (−7.0–0.5)	−9.0 (−16.5−−5.0)	< 0.001
Psychiatric work experience			
positive	−2.0 (−6.5–0.0)	−9.0 (−16.0−−4.0)	0.024
negative	−3.0 (−7.0–1.0)	−9.0 (−17.8−−5.3)	< 0.001
Frequency of medication counseling for schizophrenia patients			
Almost every day	–	–	–
A few patients a week	−3.0 (−4.0–2.0)	−9.0 (−18.0–2.0)	0.26
A few patients a month	−3.5 (−7.8–1.0)	−9.0 (−12.0−−4.0)	0.014
A few patients a year	−2.5 (−10.0−−0.25)	−9.0 (− 21.0−−5.0)	0.035
Nothing	−3.0 (−6.0–5.0)	−9.0 (−35.0−−7.0)	0.017

The values in the table represent changes in the median (inter-quartile range) total SSCP score

Statistical analysis was performed using the Mann-Whitney U test

## Discussion

The role of community pharmacists in Japan is becoming an important challenge because the number of outpatients with schizophrenia is expected to steadily increase in the future. However, the stigma of community pharmacists towards schizophrenia is a major barrier to medication support for these patients, and it also keeps pharmacists themselves from making the most of their professional skills. To the best of our knowledge, the present study is the first to develop a scale to specifically assess the stigma of schizophrenia among community pharmacists in Japan. The SSCP is unique in that it enables comprehensive characterization of “social distance at professional pharmacy service (factor I)” of community pharmacists. Specifically, it includes items that specifically address stigma among community pharmacists such as 1) if a patient hands me a prescription that includes medications for schizophrenia, I would try to avoid discussing his/her illness as much as possible. and 2) if possible, I would rather avoid home visits for patients with schizophrenia. These items are specific to scenarios that community pharmacists may face when dealing with patients diagnosed with schizophrenia; therefore, conventional scales [20] that are designed for general healthcare professionals may be insufficient to address stigma among community pharmacists. By achieving Cronbach’s *α* of 0.7 or higher for the entire scale, SSCP had sufficient internal consistency and appears to be a reliable and valid scale for assessing the stigma of schizophrenia among community pharmacists. Furthermore, a randomized controlled trial design was adopted in the present study and confirmed the effects of contact-based educational interventions using SSCP to reduce the stigma of schizophrenia among community pharmacists. A previous study reported that the recurrence rate was approximately 5-fold higher among patients diagnosed with schizophrenia who discontinued their medication than among adherent patients [25]. The stigma of schizophrenia among pharmacists negatively affects medication-related behaviors by patients, which may lead to worse symptoms due to medication withdrawal [26, 27].

Many community pharmacists at work in Japan keep their social distance from people with mental disorders [13], and concerns have been expressed over stigmatization by pharmacists, which increases the difficulties associated with appropriately managing these patients.

In pharmacotherapy for schizophrenia, for which poor medication adherence is regarded as problematic [27, 28], community pharmacists need to continuously provide medication support, including confirmation of the therapeutic and adverse effects of drugs, which helps patients to continue their medication and improve their quality of life in a responsible manner. The total SSCP and 4 factor scores of 115 pharmacists who participated in the educational program as part of the present study were lower than the midpoints. Thus, participants were individuals with relatively positive attitudes towards patients diagnosed with schizophrenia. Moreover, their values were similar to those in our previous study to develop SSCP, involving 822 community pharmacists.

In comparisons of the 2 groups (educational lecture and contact-based intervention) based on the results of the randomized controlled trial, SSCP scores improved more in the contact-based intervention group, supporting the usefulness of the educational program combining educational interventions based on knowledge of schizophrenia and communication with patients diagnosed with schizophrenia to reduce the stigma of this disorder among community pharmacists. The educational lecture on schizophrenia itself also effectively reduced this stigma; however, the effect was enhanced by adding communication with patients diagnosed with schizophrenia to the lecture. The effectiveness of an educational intervention to reduce the stigma of mental disorders has already been confirmed in meta-analyses performed in other countries [16]. Social contact or contact-based interventions have been identified as the most effective strategy [15]. Griffiths et al. previously reported that the effects of social contact to reduce stigma is enhanced by combining it with knowledge-based education [16], and these findings support the present results. Pettigrew and Tropp reported curricula complementing knowledge-based elements of education with social contact to effectively reduce stigma by decreasing anxiety and promoting empathy [29].

The present study adopted an unconventional program to provide interventions for pharmacists, with a focus on communication with patients diagnosed with schizophrenia. In this program, pharmacists initially held group discussions to clarify the difficulties associated with managing patients diagnosed with schizophrenia in daily services, and then classified their questions about these patients to directly ask them to actual patients for confirmation during a communication session for each group. Some of the questions from pharmacists, such as “How would you describe the stigmatic attitudes, statements, and behaviors any pharmacists have ever shown towards you?”, “How did you feel when you actually perceived any stigmatization by pharmacists?”, and “What do you expect from pharmacist?”, highlighted the issue of stigma, and patients freely answered these questions. At the end of the program, the contact-based intervention group discussed approaches to be adopted by pharmacists in the future based on their experience of communicating with patients diagnosed with schizophrenia.

This educational intervention content was originally provided in this program, and its usefulness was confirmed by the stronger stigma-reducing effect achieved in the contact-based intervention group than in the educational lecture group. In any case, there is currently no other educational program that specifically addresses the stigma of schizophrenia among community pharmacists in Japan or other countries.

Among the SSCP subscale scores, <factor II: attitudes towards patients diagnosed with schizophrenia > improved the most, possibly contributing to the improvements observed in scores for <factor I: social distance at professional pharmacy service >, which prevents pharmacists from making the most of their professional skills.

In our previous study, the feasibility of reducing the social distance of community pharmacists from patients diagnosed with schizophrenia by resolving their misunderstanding of these patients as dangerous was also suggested [30]. Furthermore, the establishment of an equal relationship between healthcare professionals and patients through contact-based education has been reported to contribute to reducing this social distance [31]. Community pharmacists communicate with patients diagnosed with schizophrenia in the context of a healthcare professional-patient relationship in regular pharmacy services, whereas they do so in the context of closer, person-to-person relationships in contact-based education.

Regarding the management of people with mental disorders, community pharmacists do not perceive any difficulty in providing explanations of prescriptions (such as the drug name, dosage, and dose) for these patients or explaining and confirming drugs representing their medication status; however, they encounter greater difficulties in confirming the living conditions, status/views of work (school activity), and daily life activities of these patients, such as monitoring their subjective symptoms and side effects and providing coping support [3, 32]. Pharmacists who participated in the communication session during the educational program previously held group discussions and reflected on their previous experiences of the management of patients diagnosed with schizophrenia to classify their questions to directly ask these patients and be resolved. By freely exchanging opinions beyond the boundary between patients and healthcare professionals during the communication session after this work, pharmacists may have been able to clarify their questions about patients diagnosed with schizophrenia, which may be more difficult to resolve in regular pharmacy service, and this may have consequently resolved their misunderstanding of these patients and reduced their social distance at professional pharmacy service. On the other hand, no significant differences were observed in the scores for 2 SSCP factors: <factor III: self-disclosure> and < factor IV: social distance in personal >, between the educational lecture and contact-based intervention groups. This may have been because compared with factors I and II, which represent patient factors, factors III and IV are more personal. Healthcare providers, including community pharmacists, may be making a distinction between their professional and personal attitudes. Among 115 community pharmacists that participated in the randomized controlled trial, 96 had previously come in contact with patients diagnosed with schizophrenia (personally and/ or professional), whereas the remaining 19 had no experience. Prior to the start of the educational program, the median (interquartile range) total SSCP score was 73.5 (66.0–80.0) and 75.0 (71.0–82.0) for those with and without exposure to patients diagnosed with schizophrenia, respectively. This demonstrated that exposure to patients diagnosed with schizophrenia had no significant effect on the baseline SSCP score (*p* = 0.198). We also compared the effects of the educational intervention in the contact-based intervention group (*n* = 59) based on their exposure to patients diagnosed with schizophrenia. Although the intervention significantly decreased the score compared with pre-intervention (with exposure (*n* = 51, − 9.0 (− 16.0 −− 5.0), *p* = 0.001; without exposure (*n* = 8, − 12.5 (− 36.0−− 7.0), *p* = 0.001), there was no significant difference between the 2 groups (*p* = 0.28). This suggested that the educational program was effective irrespective of the exposure of community pharmacists to patients diagnosed with schizophrenia. However, when compared between the educational lecture and contact-based intervention groups, contact-based intervention had no added effect for community pharmacists with exposure to patients diagnosed with schizophrenia. This lack of additional effect may have been because these pharmacists likely had exposure to patients diagnosed with schizophrenia that was equivalent to, or more involved than, the single contact-based intervention. Thus, the educational program may need to be revised for those with prior exposure to patients diagnosed with schizophrenia. In addition, no significant improvements were observed in the scores for <factor IV: social distance in personal > after the educational program. The reason for this currently remains unclear; however, it may be necessary to reconsider the number of SSCP factors and their contents. Regarding the relationship between pharmacies, backgrounds, and SSCP scores, there was no additional effect of communication with patients diagnosed with schizophrenia in pharmacists aged 40 or older and those who frequently communicated with patients diagnosed with schizophrenia at work and/or in their private life, suggesting that the educational program is more effective for pharmacists aged 20–39 years or with negligible experience of this form of communication. Since only limited empirical data on the influence of personal characteristics (such as sex and age) on anti-stigma education are available, further research is needed.

The present study has a number of limitations. The lecture was attended by everyone who participated in the educational program and mainly focused on biomedical knowledge about schizophrenia. The present educational lecture mainly consisted of the disease concept, causes, pathophysiology, various symptoms of each patient, cognitive functions, effects on social activities, diagnostic criteria, treatment methods, and treatment response/prognosis of schizophrenia and did not cover various psychoeducational and psychosocial aspects of mental health literacy. Such topics may trigger different responses among attendees. For example, a meta-analysis of studies on the general population demonstrated that educational interventions that focus on biomedical knowledge increased stigma among the public [33]. On the other hand, studies on health professionals demonstrated that such interventions reduced stigma [34, 35]. We found that a single lecture was sufficient for reducing stigma among community pharmacists and additional contact-based interventions further increased the effect. A previous study also reported that lectures may be effective in reducing stigma among the public if they provide information about both biomedical knowledge and effective treatment strategies for psychiatric disorders [36]. The lecture provided as part of our educational program also included information about effective treatment strategies, which may have reduced any negative impact on stigma among the attendees. The concept of psychiatric disorders, including schizophrenia, and their diagnostic criteria have markedly changed [37]. In the future, the lecture should be improved and evaluated regarding the diversity of biomedicine in schizophrenia and specific methods for helping patients undergo treatment independently, e.g., addressing problems and difficulties resulting from illnesses and disabilities and utilizing social resources. Furthermore, in the present study, SSCP was developed to create an evaluation scale specialized for community pharmacists. However, in fact, only two items in Factor I were included as questions specialized for community pharmacists. In the future, items related to stigma, characteristic of community pharmacists, should be increased. The stigma-reducing effect was only evaluated immediately after the educational intervention. The duration of this effect of contact-based educational interventions varies among studies, from immediately [38] to 12 months [39] after the intervention. Therefore, future studies are needed to examine the duration of the effects of contact-based educational interventions for community pharmacists in Japan as well as the effectiveness of repeated interventions. Moreover, further studies are required to clarify actual changes in the behavior of and daily services provided by pharmacists after participating in an educational program.

## Conclusion

The present results suggested the usefulness of our original scale of SSCP and educational program for community pharmacists with a focus on communication with patients diagnosed with schizophrenia to assess and reduce, respectively, the stigma attached to schizophrenia by these pharmacists.

## Data Availability

The datasets used and/or analyzed during the current study are available from the corresponding author on reasonable request.

## Abbreviations

SSCP: Stigma Scale towards Schizophrenia for Community Pharmacists
WSDS: the Whatley Social Distance Scale
IATM: Index of Attitudes toward the Mentally Ill
